# Seroprevalence of SARS-CoV-2 antibodies in patients with hematological and oncological diseases in early 2024

**DOI:** 10.1007/s44313-025-00067-5

**Published:** 2025-03-28

**Authors:** Louise M. Cremer, Jannik Stemler, Rosanne Sprute, Sebastian Herrmann, Theresa Markus, Jon Salmanton-García, Lutz Gieselmann, Veronica Di Cristanziano, Henning Gruell, Oliver A. Cornely, Sibylle C. Mellinghoff

**Affiliations:** 1 University of Cologne, Faculty of Medicine and University Hospital Cologne, Institute of Translational Research, Cologne Excellence Cluster on Cellular Stress Responses in Aging-Associated Diseases (CECAD), Cologne, Germany; 2 University of Cologne, Faculty of Medicine and University Hospital Cologne, Department I of Internal Medicine, Center for Integrated Oncology Aachen Bonn Cologne Duesseldorf (CIO ABCD) and Excellence Center for Medical Mycology (ECMM), Cologne, Germany; 3 German Centre for Infection Research (DZIF), Partner Site Bonn-Cologne, Cologne, Germany; 4 University of Cologne, Faculty of Medicine and University Hospital Cologne, Clinical Trials Centre Cologne (ZKS Köln), Cologne, Germany; 5 University of Cologne, Faculty of Medicine and University Hospital Cologne, Institute of Virology, Cologne, NRW Germany

**Keywords:** COVID-19, SARS-CoV-2, Vaccination, Immunocompromised patients, Communicable disease, Seroprevalence

## Abstract

**Introduction:**

COVID-19 remains a major threat to immunocompromised individuals. The determination of circulating SARS-CoV-2 antibodies in patients at high risk for severe course of SARS-CoV-2 infection is important for estimating the vaccine-induced humoral immune response. Therefore, we assessed the status quo after winter to analyze the need for booster vaccinations.

**Methods:**

Anti-spike IgG levels of 46 hospitalized patients with hematological and oncological diseases, measured between 21th December 2023 and 8th February 2024, were compared between subgroups of patients. Demographic data, underlying diseases, antineoplastic treatment, and the number of positive SARS-CoV-2 tests at the University Hospital Cologne were collected.

**Results:**

Patients with different diseases showed varying SARS-CoV-2 spike antibody levels. The highest levels were found in patients with diffuse large cell B-cell lymphoma (DLBCL) and acute leukemia who had not received specific treatment or had just initiated treatment, whereas the lowest levels were found in patients with DLBCL, acute leukemia, and multiple myeloma who had received at least one line of treatment. The geometric mean antibody titers were higher in female patients than in male patients and were highest in patients aged 41–50 years while lowest in those aged 61–70 years.

**Conclusion:**

The data presented confirm broad variations in SARS-CoV-2 anti-spike IgG levels across patients with different hematological and oncological diseases and highlight the complex interference of cancer biology, immune dysfunction, and treatment-related factors in shaping immune responses. Further research is needed to elucidate the mechanisms underlying these variations in antibody levels. We emphasize the need for regular booster vaccinations in this patient group.

## Introduction

The Coronavirus Disease 2019 (COVID-19) pandemic, caused by severe acute respiratory syndrome coronavirus 2 (SARS-CoV-2), poses unique challenges to healthcare systems worldwide. Among vulnerable populations, individuals with hematological and oncological diseases have drawn particular attention because of their immunocompromised status, which predisposes them to severe diseases following SARS-CoV-2 infection [1–4]. To determine circulating antibodies in the hematological and oncological population in seroprevalence studies and understand their role and implications for anti-SARS-CoV-2 infection as protection is crucial for guiding vaccination schedules and public health strategies to mitigate the risk of infections at the population level and to prevent infections at the individual level. With the emergence of new variants and the potential waning of vaccine-induced immunity, as well as active cancer treatment, the evaluation of anti-SARS-CoV-2 antibody levels can provide insights into the need for additional booster vaccinations and their implications for enhancing protective immunity in this vulnerable population [5, 6].

## Methods

We conducted a seroprevalence study in the winter of 2024. A total of 83 hospitalized patients with hematological and oncological diseases were screened. Of these, anti-spike-IgG levels were measured in 46 patients between 21st December 2023 and 08th February 2024, and were included in the seroprevalence analysis. Besides the anti-spike IgG level, the underlying disease and previous immunocompromising therapies were obtained and analyzed.

The following clinical data were obtained from electronic patient records: sex, age, anti-spike IgG value, underlying hematological and oncological diseases, antineoplastic treatment, number of treatment lines, and current status of the underlying disease.

The underlying diseases were categorized into the following groups: acute leukemia, diffuse large B-cell lymphoma (DLBCL), carcinoma, chronic lymphocytic leukemia (CLL), Hodgkin lymphoma, multiple myeloma, sarcoma, and other aggressive lymphoma (excluding DLBCL). Acute myeloid leukemia (AML) and acute lymphocytic leukemia (ALL) were classified as acute leukemia. DLBCL was categorized separately because of the large number of patients who were treated. Hodgkin Lymphoma includes classic Hodgkin lymphoma and nodular lymphocyte-predominant Hodgkin lymphoma (NLPHL). For solid tumors, only data from patients with high-risk chorionic carcinoma and Ewing’s sarcoma were available. Primary cerebral B-cell lymphoma, Burkitt lymphoma, angioimmunoblastic T-cell lymphoma, T-lymphoblastic lymphoma, and mature T-cell lymphoma were classified as aggressive. Myelodysplastic syndrome, hemophagocytic lymphohistiocytosis, and immune thrombocytopenia were assessed.

Humoral immunity against SARS-CoV-2 has been measured as part of clinical care. Anti-spike antibodies were measured using the LIAISON SARS-CoV-2 TrimericS IgG test on a LIAISON XL (DiaSorin, Vicenza, Italy). The assay was performed according to the manufacturer’s instructions.

The statistical parameters and applied tests are included in the respective figure legends. Statistical analyses were performed using Excel (Version 2018) and IBM SPSS v29 (SPSS, IBM Corp, Chicago, IL, United States). The tables and figures were prepared using Microsoft Excel and Flourish. Data were analyzed anonymously, therefore our institutional review board waived the necessity of informed consent (No. 24–1120-retro).

Results were correlated with the number of SARS-CoV-2 infections measured at the University Hospital Cologne in patients who underwent testing for SARS-CoV-2 infection within the applicable timeframe. If patients were tested more than once during the mentioned period, only the first negative test result was included and, if applicable, the first positive result was included.

## Results

We included 46 patients with different hematological and oncological diseases (Table 1). Most patients had acute leukemia (*n* = 9 [19.6%]), DLBCL (*n* = 9 [19.6%]), or multiple myeloma (*n* = 9 [19.6%]). Of 46 patients, most (*n* = 39) were actively treated for underlying hematological and oncological diseases. Seven patients were treatment-naïve and were still undergoing diagnostic procedures or shortly before treatment initiation. In total, 27 of the 46 patients (58.7%) received the first treatment line, and 12 were in advanced treatment lines (second- or later-line systemic therapy). Nearly half of the patients received B-cell-depleting treatment (*n* = 19 [41.3%]) and 15 patients received anti-CD20-antibodies (rituximab and obinutuzumab), while only one patient was treated with bruton-tyrosine-kinase (BTK) inhibitors (ibrutinib), one with B-Cell-Lymphoma-2 (BCL-2) inhibitors (venetoclax), and one with bispecific antibodies (talquetamab).

**Table 1:** Patient characteristics

Characteristic	N [%]
Sex	
Female	21 (45.7)
Male	25 (54.3)
Age categories	
21–30	5 (10.9)
31–40	7 (15.2)
41–50	5 (10.9)
51–60	12 (26.1)
61–70	11 (23.9)
> 71	6 (13.0)
Hematological and oncological disease	
Acute leukemia	9 (19.6)
CLL	2 (4.3)
DLBCL	9 (19.6)
Hodgkin Lymphoma	3 (6.5)
Multiple myeloma	9 (19.6)
Other aggressive lymphoma	8 (17.4)
Carcinoma	1 (2.2)
Sarcoma	2 (4.3)
Others	3 (6.5)
Treatment status	
Treatment naïve	7 (15.2)
Active treatment	39 (84.8)
< 2 treatment lines	27 (69.2)*
≥ 2 treatment lines	12 (30.8)*
Treatment	
B-cell depleting treatment	19 (41.3)
< 12 months	16 (84.2)*
≥ 12 months	3 (15.8)*
BCL-2-inhibitors	1 (2.2)
B-cell directed	18 (39.1)
BTKi	1 (5.6)*
Anti CD20-antibodies	15 (83.3)*
Bispecific antibodies	1 (5.6)*
Other	1 (5.6)*
Other targeted therapies	15 (32.6)
Chemotherapy	29 (63.0)
HSCT	3 (6.5)
Allogeneic	1*
Autologous	2*

*CLL* Chronic Lymphocytic Leukemia, *DLBCL* Diffuse Large Cell B-Cell Lymphoma, *HL* Hodgkin Lymphoma, *MM* Multiple Myeloma, *BTKi* Bruton-Tyrosine-Kinase-Inhibitor, *BCL2* B-Cell-Lymphoma-2, *HSCT* Hematopoietic Stem Cell Transplantation, *Allo* Allogeneic, *Auto* Autologous

^*^ % of N of the applicable group

**Fig. 1:**
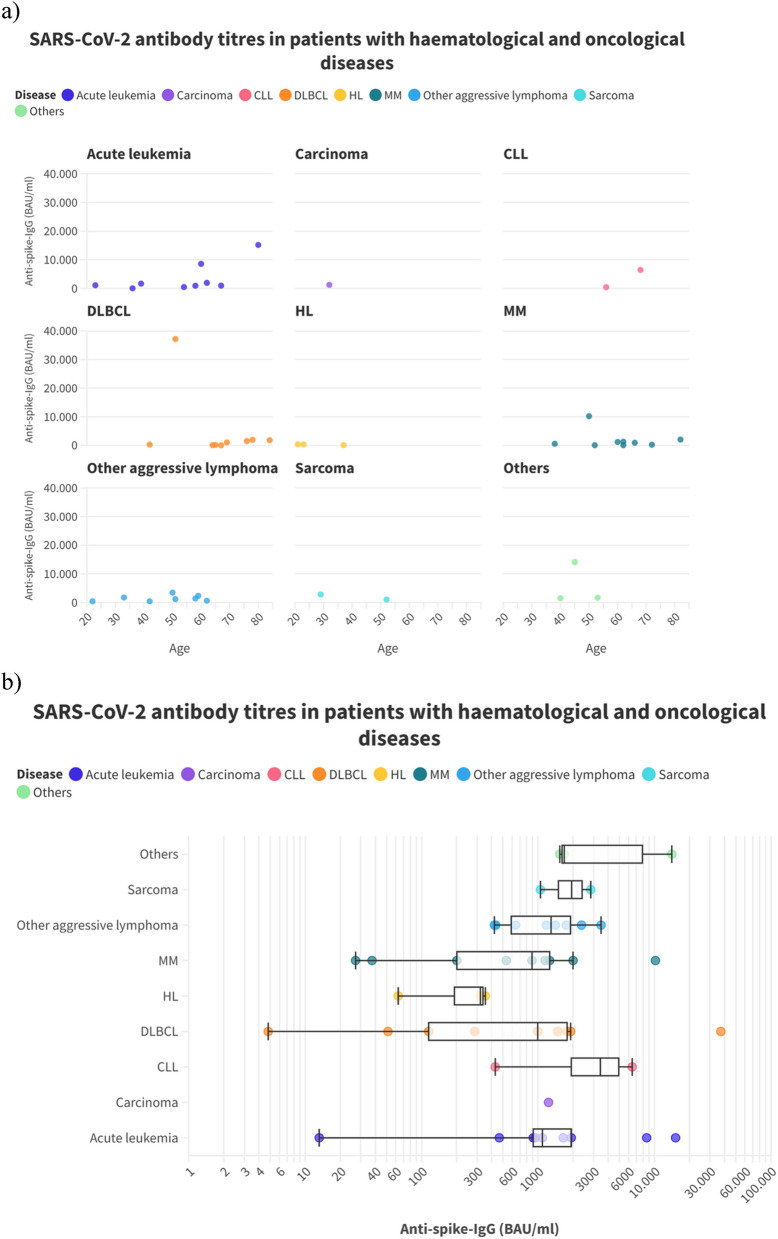
SARS-CoV-2 antibody titers in different groups of patients with hematological and oncological diseases – Scatter Plot (**a**) and box plot (**b**)

**Fig. 2:**
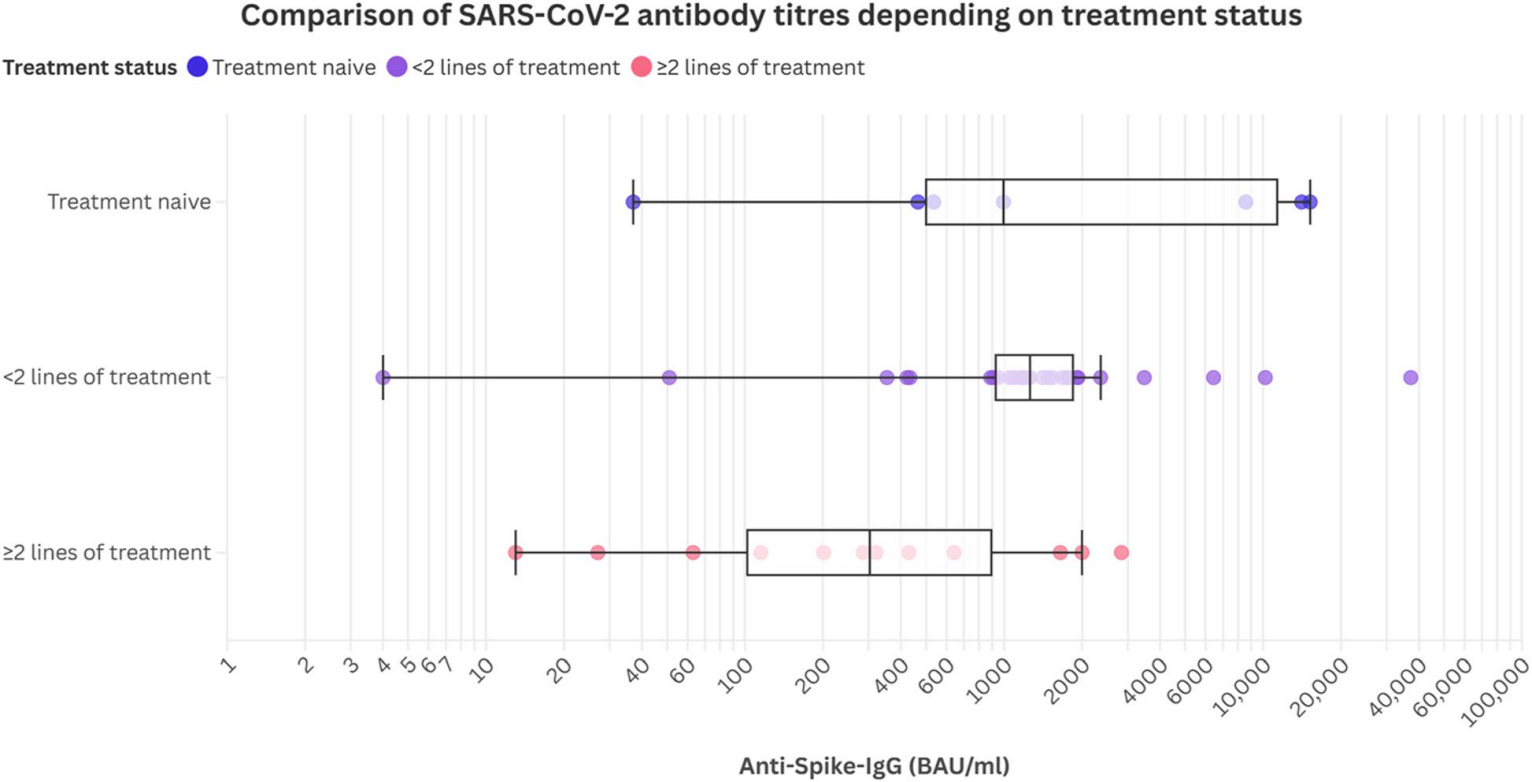
Comparison of SARS-CoV-2 antibody titers depending on treatment status

The highest levels were found in patients with DLBCL and acute leukemia who had not yet received specific treatment or had just started treatment in the week before antibody measurement, while the lowest levels were found in patients with DLBCL, acute leukemia, and multiple myelomas who had received at least one line of treatment including chemotherapy, B-cell depletion, and targeted treatment as well as autologous and allogeneic hematopoietic stem cell transplantation (HSCT) in the past (Fig. 1a/b and Fig. 2). Descriptive analysis revealed a spectrum of seropositivity rates and antibody titers among the cohorts.

Geometric mean antibody titers were higher in female patients (*n* = 21 [45.7%]; anti-spike-IgG = 975.6 BAU/ml) than in male patients (*n* = 25 [54.3%]); anti-spike IgG = 698.9 BAU/ml, *p* = 0.55).

**Fig. 3:**
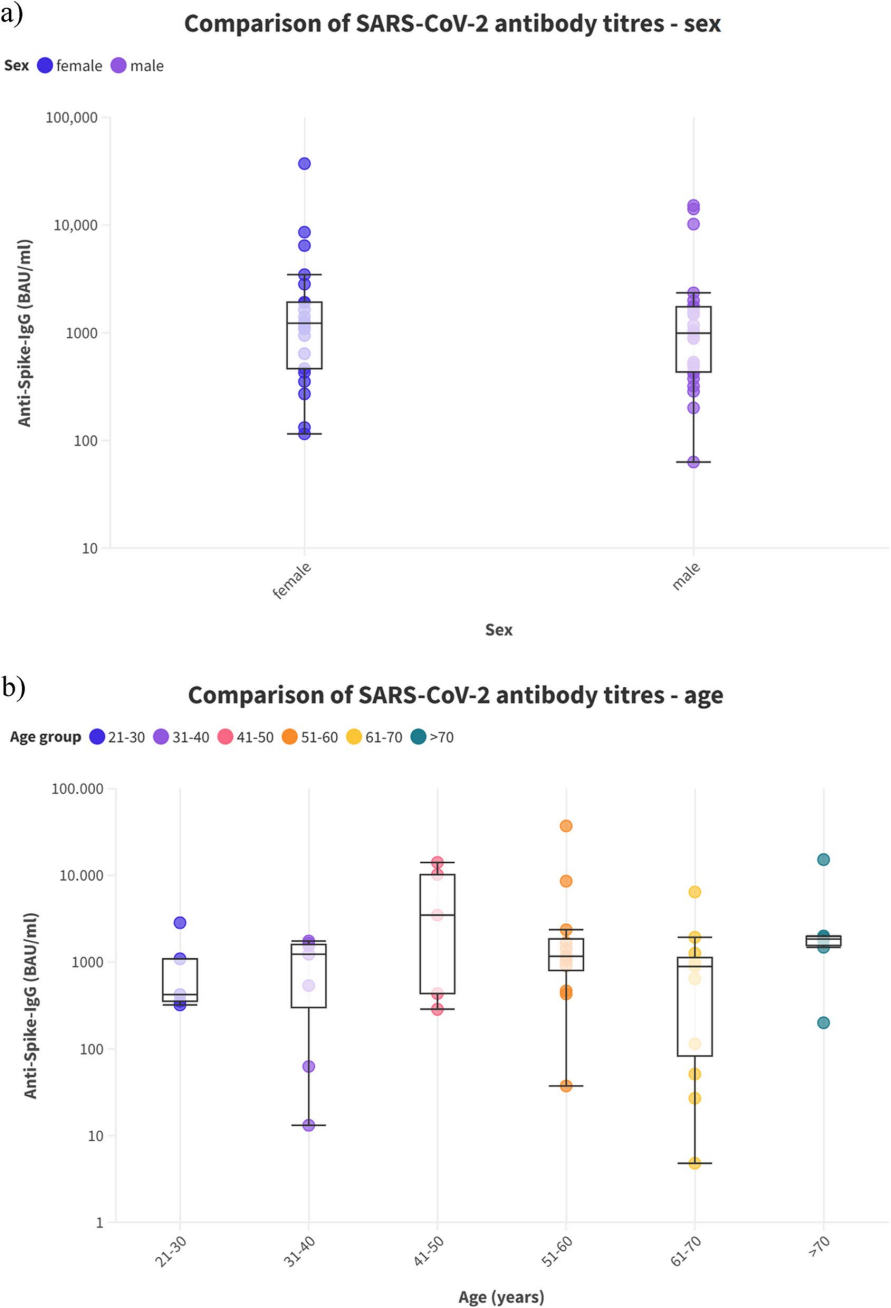
Comparison of SARS-CoV-2 antibody titers **a**) sex **b**) age

In total, geometric mean antibody titers (Fig. 3a/b) were highest in patients aged 41–50 years (*n* = 5 [10.9%]; anti-spike IgG = 2282.7 BAU/ml, *p* = 0.549) and lowest in the age group of 61–70 years (*n* = 11 [24%]; anti-spike IgG = 337.5 BAU/ml, *p* = 0.549).

**Fig. 4:**
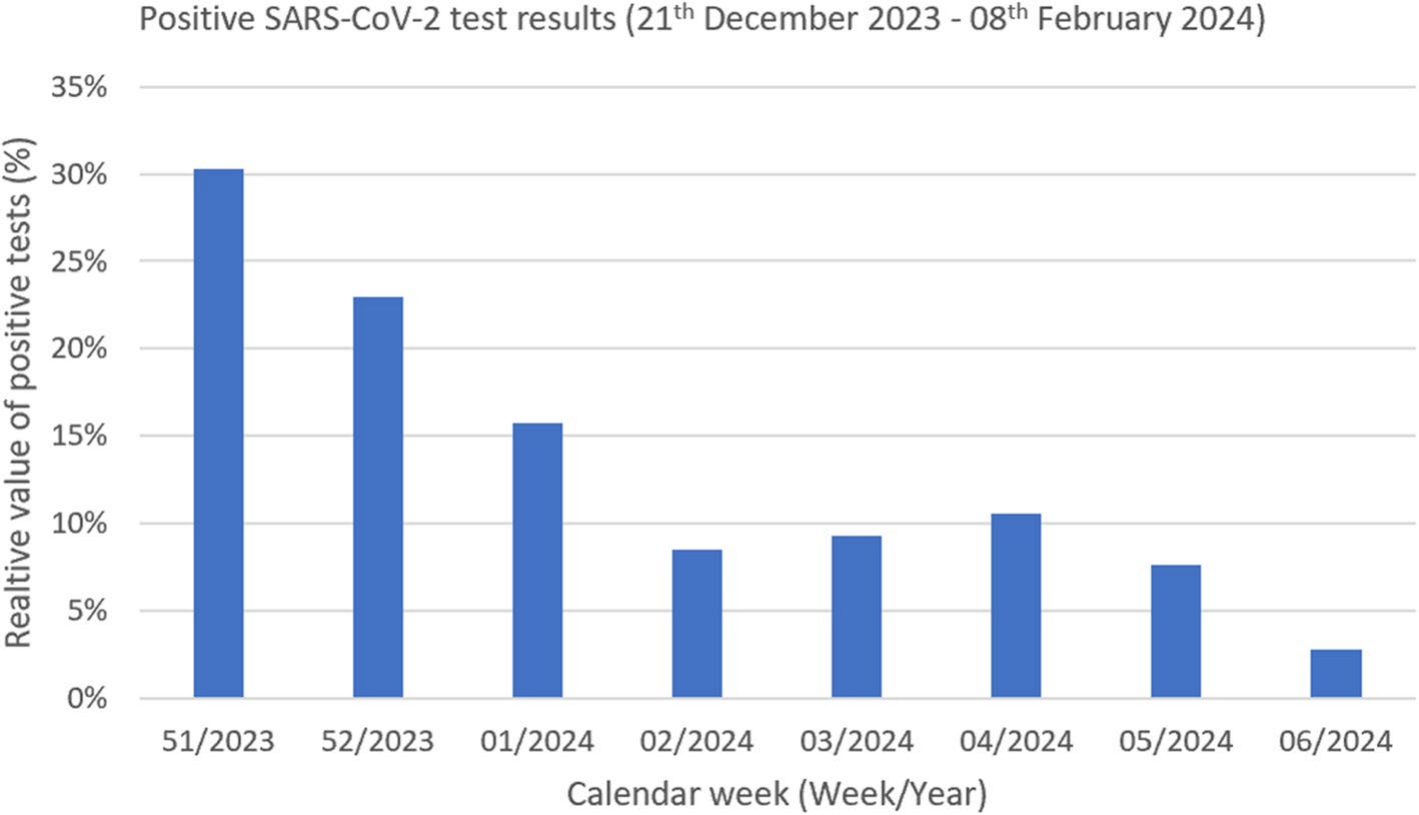
SARS-CoV-2 test results at University Hospital Cologne between December 2023 and February 2024

The number of SARS-CoV-2 tests performed at our center is shown in Fig. 4. The highest number of positive results was registered in the middle of December (51/2023), with 30% positive cases, and then declined and stagnated between 8 and 11% in January and beginning of February (02/2024 to 06/2024).

## Conclusion

In this study, we describe the antibody levels determined to assess the necessity for future booster strategies for COVID-19 vaccination.

The observed variations in antibody levels underscore the complex interplay between cancer biology, immune dysfunction, and treatment-related factors in shaping the immune responses to SARS-CoV-2 [7]. Patients with active immunocompromising treatment and two or more prior treatment lines show an impaired ability to mount effective antibody responses due to strong B-cell affection, whereas patients in first-line or without active treatment often retain partial immune function, leading to more robust antibody production. Furthermore, the impact of specific treatment modalities such as chemotherapy, immunotherapy, and stem cell transplantation on immune competence and vaccine responsiveness warrants careful consideration. We found that patients who had shortly been diagnosed with a hematological or oncological disease and had just started treatment often showed higher antibody levels. This likely reflects immune competence after prior vaccinations with persistent antibody titers despite impaired immune function due to malignancy. The highest variations in antibody levels were observed in patients with acute leukemia and aggressive lymphoma, which is consistent with previous reports [1].

Previous research has shown a correlation between heightened anti-spike IgG levels and a decreased risk of severe COVID-19. However, an exclusive Correlation of Protection has not yet been established and is challenging to define [8, 9]. A recent study found that the risk of fatal COVID-19 was inversely correlated with anti-spike IgG levels below the 20th percentile in a large cohort of 3012 nursing home residents [10]. Based on these results, the anti-spike IgG levels of participants in a previous seroprevalence study were reanalyzed to identify the 20th percentile as a cut-off value indicative of a positive vaccine response [11]. As a result, an anti-spike-IgG titer of ≥ 847 BAU/ml was defined as an adequate antibody level. Only 29 of 46 patients (63%) in this analysis reached antibody levels ≥ 847 BAU/ml, of which 14 (48%) were women.

Higher antibody levels were observed in female than in male patients. Higher vaccine responses have previously been reported in female children and adults, i.e., higher and longer-lasting levels of vaccine-specific IgM and IgG. In addition, more adverse events linked to an increased immune response have been reported in female patients following vaccination, including the COVID-19 vaccination [12, 13]. Age being a prominent risk factor for reduced humoral vaccine-induced immunity, was not correlated with low antibody titers in this analysis indicating the likely higher relevance of immunosuppression as influencing factor in this setting [14].

Considering that the incidence of SARS-CoV-2 infection peaks in December, we suggest booster vaccination with the most recently licensed and WHO-approved COVID-19 vaccine for hematological and oncological patients at the beginning of autumn.

Tailored approaches to vaccination, including personalized booster strategies or alternative immunization regimens, may be necessary to optimize the protective immunity in this heterogeneous population. Previously, it was shown that repeated booster vaccinations yield the potential for a continuous increase of the humoral [15] and, probably, even the T cell immune response [16]. Ongoing clinical trials, such as the Auto-COVID-VACC study conducted by the University of Cologne, are evaluating the humoral and cellular immune responses in immunocompromised patients receiving up to eight COVID-19 vaccinations depending on their individual antibody responses [17].

Apart from investigating humoral and cellular immune responses, attention must be paid to vaccinating at-risk patients and their household members. The current recommendations of the Robert-Koch-Institue (RKI) [18] and the Infectious Diseases Working Party (AGIHO) of the German Society for Hematology and Medical Oncology (DGHO) [2] are a minimum of three antigen contacts (vaccination or infection), of which at least one should be by vaccination. Further booster vaccinations are recommended for people with immunodeficiencies, such as patients with hematological and oncological diseases, and should be administered at least four weeks after the last vaccination. The RKI, as well as the AGIHO, mention the possibility of antibody-level testing, however, they show its limitations. For example, the cellular immune response may differ and is not considered when measuring antibody levels. Furthermore, there is still no recommendation for a sufficient antibody level for protection from infection, which should be taken into account when deciding whether further booster vaccine doses are required, indicating the need for further research in this area [2, 18]. We report vaccination rates of 95% regarding COVID-19 vaccination (Q3/4 2023) in our patients (data unpublished). However, considering the experience with other seasonal vaccines (e.g., influenza vaccine rates of 44.6% in our patients; unpublished data), we expected a decline in these high vaccination rates. Current analyses by the RKI show that 73,4% of the German population aged over 18 years has completed priming immunization and 19,4% of the adult population has received two booster vaccine doses after completing basic immunization [19]. Therefore, vaccination programs must alert patients and their treating physicians of booster vaccinations along current recommendations.

In addition to antibody levels, the role of T-cell responses in patients with hematological and oncological diseases following SARS-CoV-2 infection or vaccination warrants attention. The measurement of antibodies alone is insufficient to estimate vaccine-induced immune responses. While antibodies are critical components of the immune response, mounting evidence suggests that T cell-mediated immunity also plays a pivotal role in controlling viral infections, including COVID-19 [20]. Assessing T-cell responses alongside antibody levels is essential for comprehensively evaluating vaccine-induced immune responses in this population and is the subject of ongoing studies. Relying solely on antibody levels may lead to underestimation of vaccine-induced immune protection, particularly in immunocompromised populations [21].

This study had several limitations. First, the correlation between anti-spike IgG values and the last infection or vaccination, as well as the number of vaccine doses received, was not analyzed, however, it might have an impact on varying antibody levels across the study cohort. Second, the study cohort was too small to identify specific treatment regimens, leading to varying antibody levels. Furthermore, only anti-spike-IgG values were analyzed here, however, further laboratory tests analyzing humoral and cellular immune responses may also provide further explanations for the varying antibody levels in patients with hematological and oncological diseases.

The data presented here show broad variations in SARS-CoV-2 anti-spike antibody levels across different hematological and oncological diseases, highlighting the complex interference of cancer biology, immune dysfunction, and treatment-related factors in shaping immune responses to SARS-CoV-2. Further research is needed to elucidate the underlying mechanisms driving variations in antibody levels among different hematological and oncological diseases, and to evaluate the durability of vaccine-induced immunity in this context. Longitudinal studies assessing antibody kinetics over time and correlating antibody responses with clinical outcomes will provide valuable insights into the effectiveness of current vaccination strategies and the need for future booster vaccinations tailored to the unique needs of patients with various hematological and oncological diseases.

## Data Availability

Data available on request from the authors. The data that support the findings of this study are available from the corresponding author upon reasonable request. Basic, Share upon Request.

## Abbreviations

ALL: Acute Lymphocytic Leukemia
Allo: Allogeneic
AML: Acute Myeloid Leukemia
Auto: Autologous
AGIHO: Infectious Diseases Working Party of the German Society for Hematology and Medical Oncology
BAU: Binding Antibody Units
BTKi: Bruton-Tyrosine-Kinase-Inhibitor
BCL2: B-Cell-Lymphoma-2
CLL: Chronic Lymphocytic Leukemia
COVID-19: Coronavirus Disease 2019
DGHO: German Society for Hematology and Medical Oncology
DLBCL: Diffuse Large B-Cell Lymphoma
HL: Hodgkin Lymphoma
HSCT: Hematopoietic Stem Cell Transplantation
IgG: Immunoglobulin G
MM: Multiple Myeloma
NLPHL: Nodular lymphocyte-predominant Hodgkin lymphoma
RKI: Robert-Koch-Institute
SARS-CoV-2: Severe acute respiratory distress syndrome
